# The Potential of CRISPR/Cas9 Gene Editing as a Treatment Strategy for Inherited Diseases

**DOI:** 10.3389/fcell.2021.699597

**Published:** 2021-12-15

**Authors:** Sameh A. Abdelnour, Long Xie, Abdallah A. Hassanin, Erwei Zuo, Yangqing Lu

**Affiliations:** ^1^ State Key Laboratory for Conservation and Utilization of Subtropical Agro-Bioresources, College of Animal Science and Technology, Guangxi University, Nanning, China; ^2^ Animal Production Department, Faculty of Agriculture, Zagazig University, Zagazig, Egypt; ^3^ Center for Animal Genomics, Agricultural Genome Institute at Shenzhen, Chinese Academy of Agricultural Sciences, Shenzhen, China; ^4^ Genetics Department, Faculty of Agriculture, Zagazig University, Zagazig, Egypt

**Keywords:** CRISPR/Cas9, gene editing, rectifying, genetic diseases, treatment

## Abstract

Clustered regularly interspaced short palindromic repeats (CRISPR) is a promising innovative technology for genomic editing that offers scientists the chance to edit DNA structures and change gene function. It has several possible uses consisting of editing inherited deficiencies, treating, and reducing the spread of disorders. Recently, reports have demonstrated the creation of synthetic RNA molecules and supplying them alongside Cas9 into genome of eukaryotes, since distinct specific regions of the genome can be manipulated and targeted. The therapeutic potential of CRISPR/Cas9 technology is great, especially in gene therapy, in which a patient-specific mutation is genetically edited, or in the treating of human disorders that are untreatable with traditional treatments. This review focused on numerous, *in vivo*, *in vitro,* and *ex vivo* uses of the CRISPR/Cas9 technology in human inherited diseases, discovering the capability of this versatile in medicine and examining some of the main limitations for its upcoming use in patients. In addition to introducing a brief impression of the biology of the CRISPR/Cas9 scheme and its mechanisms, we presented the utmost recent progress in the uses of CRISPR/Cas9 technology in editing and treating of human genetic diseases.

## Overview of CRISPR/Cas9 toolbox

Medicating diseases through genetic modifications or even attaining the capability to repair any portion of the organism’s genome precisely and persistently has long been an aspiration for researchers (Yin et al., 2014). In the last few years, the precipitous progress of genome editing tools has started up new opportunities in the field of medical sciences. Gene editing as a versatile technique in the curing of several disorders has made considerable advancements, as well as substantially expanding the capability to rectify and motivationally manipulate the eukaryotic cell genome (Cox et al., 2015; Syding et al., 2020). There are a variety of high-accuracy gene editing tools to remedy various hereditary disorders. The clustered regularly interspaced short-palindromic repeat (CRISPR)-associated protein 9 (CRISPR/Cas9) nuclease scheme has appeared as a persuasive genome manipulating invention for several agricultural, biological, and biomedical proposes (Cong et al., 2013; Yin et al., 2014; Long et al., 2016). The CRISPR/Cas9 technology has shown to be a potent innovative approach that authenticates on Cas9/sgRNA ribonucleoprotein complexes (RNPs) to intend and manage DNA sequence. This approach is deemed a highly versatile and potent technology both for transcriptional manipulating and gene editing as well as for epigenetic modulation. In the era of post-genomics, CRISPR/Cas9 technology has been employed to explore intended genes in genome splicing (Yuan et al., 2018), transcription (Abudayyeh et al., 2017), modification (Oakes et al., 2019), and epigenetic regulation (Xie et al., 2018). However, CRISPR/Cas9-mediated HDR is ineffective and may present deleterious off-target double-stranded breaks (dsDNA), hindering possible clinical uses (Kosicki et al., 2018; Geurts et al., 2020). Recently, the developed Cas9 fusion proteins, so-termed base editors (BE), could manage and avoid these topics. Recent studies demonstrated the fusion of a cytidine deaminase to a partially inactive nickase Cas9 protein permits for competent C-G to T-A base changes (C-T base editing), whereas a fusion with a changed TadA heterodimer achieves the contradictory response, from A-T to G-C (A-G) (Koblan et al., 2018; Zafra et al., 2018). This developed strategy BE acts only within a small window of the single-stranded R-loop that is produced upon Cas9 binding to the target sequence. Moreover, among the spectacular developments, the translational application of CRISPR/Cas9 in different human hereditary disorders can offer long-term treatment after a single medication. Several human hereditary disease models have been produced employing the versatile CRISPR/Cas9 technology, as well as the genetic therapy of some selected genetic disorders *in vitro* and *in vivo*, which are presented in Table1 (summarized in Table 1).

**TABLE 1:** Preclinical CRISPR/Cas9 therapy in inherited disease models presented in the current article.

Disease	Target gene	Model (animal or cell)	Findings	References
β-thalassemia	*HBB*	CD34+ HSPCs of β-thalassemia patients	▪ 93.0% indel frequency (SpCas9)	Xu et al. (2021)
	*KLF1*	human myeloid leukemia (K562) cell line	▪ The average indel percentage in the cells transfected with CRISPR was nearly 24%	Shariati et al. (2016)
			▪ Relative quantification was performed for the assessment of γ-globin expression	
			▪ The levels of γ-globin mRNA on day 5 of differentiation were significantly higher in the cells treated with CRISPR/Cas9 compared to untreated cells	
Atherosclerosis	*LDLR*	Homogeneous *Ldlr* ^*E208X*^ mice	▪ *In vivo* AAV-CRISPR/Cas9–mediated *Ldlr* gene correction can partially rescue LDLR expression and effectively ameliorate atherosclerosis phenotypes in *Ldlr* mutants	Zhao et al. (2020)
			▪ Compared with the control, the AAV-CRISPR/Cas9 with targeted single guide RNA group had significant reductions in total cholesterol, total triglycerides, and LDL cholesterol in the serum, whereas the aorta had smaller atherosclerotic plaques and a lower degree of macrophage infiltration.	
Phenylketonuria	Pah	Pah^*enu2/enu2*^ mouse model	▪ Permanent correction of the Pahenu2 allele in a portion of treated hepatocytes of mice with PKU, yielding partial restoration of liver PAH activity, substantial reduction of blood phenylalanine, and prevention of maternal PKU effects during breeding	Richards et al. (2020)
	*Pah*	*PAH*-null pigs	▪ After selection of optimal CRISPR/Cas9 genome-editing reagents by using an *in vitro* cell model, zygote injection of 2 sgRNAs and Cas9 mRNA demonstrated deletions in preimplantation embryos, with embryo transfer to a surrogate leading to 2 founder animals	Koppes et al. (2020)
			▪ One pig was heterozygous for a PAH exon 6 deletion allele, while the other was compound heterozygous for deletions of exon 6 and of exons 6–7. T	
			▪ The affected pig exhibited hyperphenylalaninemia (2000–5000 μM) that was treatable by dietary Phe restriction, consistent with classical PKU, along with juvenile growth retardation, hypopigmentation,ventriculomegaly, and decreased brain gray matter volume	
Facioscapulohumeral muscular dystrophy (FSHD)	*DUX4*	treating patient cells and zebrafish models	▪ Application of hypoxia signaling inhibitors resulted in increased DUX4 protein turnover and subsequent reduction of the cellular hypoxia response and cell death	Lek et al. (2020)
			▪ In addition, these compounds proved successful in reducing FSHD disease biomarkers in patient myogenic lines, as well as improving structural and functional properties in two zebrafish models of FSHD	
Duchenne musculardystrophy (DMD)	*Dmd*	*mdx* ^*4cv*^ mice	▪ Treated muscles express dystrophin in up to 70% of the myogenic area and increased force generation following intramuscular delivery	Bengtsson et al. (2017)
	*Dmd*	HEK293T cells	▪ The unique multiplex gene editing capabilities of the CRISPR/Cas9 system facilitate the generation of a single large deletion that can correct up to 62% of DMD mutations	Ousterout et al. (2015)
	*DMD*	mouse model	▪ A low dose of scAAV-delivered CRISPR-Cas genome editing components is sufficient to restore dystrophin protein expression, reduce DMD pathological phenotypes, and improve muscle function in a DMD mouse model	Zhang et al. (2020)
	*DMD*	*Dog*	▪ Protein expression of up to 6% of normal levels was seen in two dogs injected with sgRNA B and up to 16% of normal in one dog treated with sgRNA A. TALEN did not restore any dystrophin expression	Mata López et al. (2020)
			▪ While there were no adverse effects, clear benefits were not seen on histopathologic analysis, immunofluorescence microscopy, and force measurements	
Cystic fibrosis	*CFTR*	Domestic sheep (*Ovis aries*)	▪ The newborn CFTR–/– sheep developed severe disease consistent with CF pathology in humans	Fan et al. (2018)
			▪ Substantial liver and gallbladder disease may reflect CF liver disease that is evident in humans	
	*CFTR*	Rabbit	▪ CF rabbits exhibited human CF–like abnormalities in the bioelectric properties of the nasal and tracheal epithelia	Xu et al. (2021)
			▪ No spontaneous respiratory disease was detected in young CF rabbits	
			▪ However, abnormal phenotypes were observed in surviving 1-year-old CF rabbits as compared with WT littermates, and these were especially evident in the nasal respiratory and olfactory epithelium	
	*CFTR*	human embryonic kidney cells (HEK293, ATCC CRL-1573)	▪ Their results indicate the feasibility of site-specific gene targeting with the CRISPR/Cas9 system. 33% of the samples were corrected using CRISPR in mutant locus and confirmed by sequence blast at NCBI databases and primers outside the arm locus	Khatibi et al. (2021)
			▪ CRISPR/Cas9 approach represents an efficient tool to repair the ΔF508 mutation of the CFTR gene in PBMC Cells	
	*CFTR*	human bronchial epithelial cells	▪ Differentiated epithelial monolayers cultured at air-liquid interface showed restored CFTR function that was >70% of the CFTR function in non-CF controls	Vaidyanathan et al. (2021)
Ornithine transcarbamylase (OTC	OTC	MC57G cells And spfash mice	▪ This resulted in reversion of the mutation in 10% (6.7–20.1%) of hepatocytes and increased survival in mice challenged with a high-protein diet, which exacerbates disease	Yang et al. (2020)
			▪ Gene correction in adult OTC-deficient mice was lower and accompanied by larger deletions that ablated residual expression from the endogenous OTC gene, leading to diminished protein tolerance and lethal hyperammonemia on a chow diet	
	OTC	spfash mice	▪ Therapeutic effect of AAV-delivered, CRISPR-Cas9–mediated gene targeting in a mouse model of OTCD	Wang et al. (2020)
			▪ In the absence of any selective growth advantage for OTC-positive cells, a single injection of dual AAV gene-targeting vectors in neonatal mice achieved robust and sustained expression of OTC that was clinically beneficial	
Primary hyperoxaluria type I (PH1)	AGXT gene	Agxt1−/− mice (B6.129SvAgxt^tm1Ull^) mice	▪ A single systemic administration of an AAV8-CRISPR/Cas9 vector targeting glycolate oxidase, prevents oxalate overproduction and kidney damage, with no signs of toxicity in Agxt1−/− mice	Zabaleta et al. (2018)
	*AGXT gene*	Induced pluripotent stem cells from the PH1 patient. Hepatocyte-like cells (HLCs)	▪ The CRISPR/Cas9 nucleasee-mediated gene targeting of a single-copy AGXT therapeutic minigene into the safe harbor AAVS1 locus in PH1-induced pluripotent stem cells (PH1-iPSCs) without off-target inserts	Estève et al. (2019)
			▪ They obtained a robust expression of a codon-optimized *AGT* in HLCs derived from AAVS1 locus-edited PH1- iPSCs	
			▪ The study provides the proof of concept that CRISPR/Cas9-mediated integration of an *AGXT* minigene into the AAVS1 safe harbor locus in patient-specific iPSCs is an efficient strategy to generate functionally corrected hepatocytes	
	*Ldha*	Pheochromocytoma (PC12) cells	▪ The *Ldha* gene was specifically knocked out in 20% of the liver cells of PH1 rats in the treatment group, leading to a 50% lower LDH expression than that in the control group	Zheng et al. (2020)
			▪ Compared to the control groups, urinary oxalate levels were significantly decreased, and renal calcium oxalate precipitation was largely mitigated in the treatment group throughout the entire 6-months study period	
			▪ While no CRISPR/Cas9-associated off-target edits or hepatotoxicity were detected, we observed mild metabolic changes in the liver tricarboxylic acid (TCA) and glycolysis pathways	
	*AGXT*	Rat	▪ Mutant rats exhibited crystalluria and showed a slight dilatation of renal tubules with mild fibrosis in the kidney	Zheng et al. (2018)
			▪ Mutant rats excreted greater abundance of oxalate and developed severe nephrocalcinosis in contrast to WT animals. Significantly elevated expression of inflammation and fibrosis related genes was also detected in mutants	
Recessive dystrophic epidermolysis bullosa (RDEB)	*COL7A1*	Primary fibroblasts	▪ Gene-corrected keratinocytes exhibited characteristic epithelial morphology and expressed keratinocyte-specific genes and transcription factors	Webber et al. (2016)
			▪ Induced pluripotent stem cells -derived MSCs exhibited a spindle morphology and expression of CD73, CD90 and CD105 with the ability to undergo adipogenic, chondrogenic and osteogenic differentiation *in vitro* in a manner indistinguishable from bone marrow-derived MSCs	
	*COL7A1*	The embryonic kidney cell line HEK293. Human RDEB keratinocytes	▪ Next-generation sequencing revealed on-target efficiency of up to 30%, whereas nuclease-mediated off-target site modifications at predicted genomic loci were not detected	Hainzl et al. (2017)
	*COL7A1*	HEK293 cells *mice*	▪ CRISPR/Cas9 targeting this specific *COL7A1 gene* was delivered to recessive dystrophic epidermolysis bullosa patient fibroblasts	Takashima et al. (2019)
			▪ After genotyping a large collection of gene-edited fibroblast clones, we identified a significant number (17/50) of clones in which the frameshift in *COL7A1* was restored	
			▪ The reframed *COL7* was functional, as shown by triple-helix formation assay in vitro, and was correctly distributed in the basement membrane zone in mice	
	*COL7A1*	Primary wild-type keratinocytes immortalized RDEB keratinocytes	▪ Type VII collagen knockout in more than 40% of ribonucleoprotein-treated primary wild-typekeratinocytes and type VII collagen restoration in more than 70% of ribonucleoprotein-treated recessive DEB keratinocytes	Kocher et al. (2020)
			▪ Next-generation sequencing of the on-target site revealed the presence of the precise adenine insertion upstream of the pathogenic mutation in at least 17% of all analyzed COL7A1 alleles	
Hearing loss	*Tmc1*	Primary fibroblasts and mice	▪ Results observed higher hair cell survival rates and lower auditory brainstem response thresholds in injected ears than in uninjected ears or ears injected with control complexes that targeted an unrelated gene.	Farooq et al. (2020)
			▪ Enhanced acoustic startle responses were observed among injected compared to uninjected *Tmc1* ^*Bth*/+^ mice	
	*ARHL*	Mice	▪ Correction of the progressive hearing loss phenotype was demonstrated using auditory-evoked brainstem response testing of mice at 24 and 36 weeks of age, and rescue of the progressive loss of sensory hair cell stereocilia bundles was confirmed using scanning electron microscopy of dissected cochleae from 36-week-old mice	Mianné et al. (2016)

Base editing has been used by laboratories around the world in a wide range of organisms and cell types. By integrating base editors with *in vivo* delivery strategies, by addressing animal models of human genetic diseases such as progeria, with a high degree of phenotypic rescue and lifespan extension (Koblan et al., 2021).

Prime editing offers efficiency and product purity advantages over HDR, complementary strengths and weaknesses compared to base editing, and lower off-target editing than Cas9 nuclease at known Cas9 off-target sites. Prime editing further expands the scope and capabilities of genome editing.

A number of additional studies may further advance base-editing treatments for progeria towards clinical application. It was recently reported that ABE variants have much higher editing activity than ABE7.10max (Gaudelli et al., 2020; Richter et al., 2020). These variants could further increase editing efficiency and phenotypic rescue, or might reduce the required dosage. The timing of treatment may also need to be further optimized for best outcomes, taking into account the time to diagnosis. Finally, ABE editing has the potential to synergize with emerging progeria treatments (Beyret et al., 2019; Lai and Wong, 2020) including farnesyltransferase inhibitors (Gordon et al., 2014), other small-molecule drugs (Lai and Wong, 2020), or antisense oligonucleotides that target the mutant LMNA allele (Osorio et al., 2011).

This review will briefly introduce the history and mechanism of the CRISPR/Cas9 technology as well as some advances and innovative approaches for enhancing the efficiency of the application of CRISPR/Cas9 technology. Moreover, we will describe the potentiality of applying the CRISPR/Cas9 in the therapy of several human genetic diseases according to the *in vitro* and *in vivo* studies.

## CRISPR/CAS9: History and Mechanism

CRISPR was initially detected in *Escherichia coli* by Ishino et al. (1987) as repeats of DNA with unknown function. After, Mojica et al. (2000) determined the same kinds of repeats in other microorganisms (Jansen et al., 2002; Lander, 2016). Recently, the CRISPR sequences were found to participate in the immune system of bacteria against viruses *via* interacting and cutting some parts in the DNA sequence of the viral pathogens with Cas nucleases enzyme (Barrangou et al., 2007; Jinek et al., 2012). Interestingly, the Cas enzyme acts as pathogen-specific by a specific estate of the enzyme and the guide RNA stimulates the enzyme and guides it to the target DNA sequences. This distinguishing feature of the Cas enzyme qualified its application to generating DNA breaks at a specific site in target genomic DNA *in vivo*. Cas9 from *E. coli* bacteria is the most explored and broadly exploited among the various Cas nucleases. Due to the specificity and flexibility of DNA-sequence, the CRISPR/Cas9 technology has been subjected to lots of investigational surveys concerning invertebrates and mammals (Mali et al., 2013b; Cong et al., 2013).

That Cas9 protein cleaves the DNA at specific sites was discovered by Makarova et al. (2006), who performed a relative genomic assessment of CRISPR and Cas genes. They predicted that there were similarities between CRISPR/Cas9 technology and the function of RNA interference, hence the protein silences the gene by cleaving mRNA, while the Cas9 protein cleaves DNA. In the same context, some Cas proteins like Cas13 cleave RNA (Makarova et al., 2006).

**FIGURE 1:**
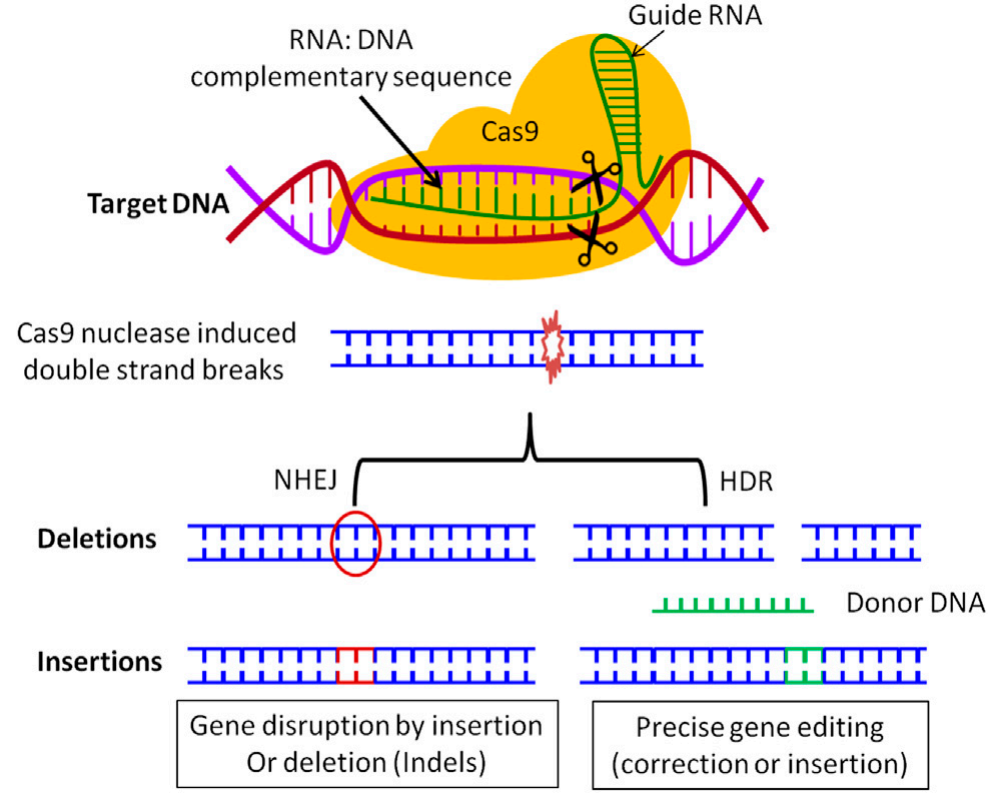
CRISPR mechanism. The Guide RNA hybridizes with the target DNA sequence and directs Cas9 endonuclease (colored in yellow) to generate a double-strand break. Subsequently, mutant DNA is generated from the repair process of DNA, through either the homology-directed repair (HDR) or the non-homologous end-joining (NHEJ) mechanism. The final mutant DNA could include deletion or insertion of DNA sequence (NHEJ), or replacement with a particular DNA sequence used as a marker for further study (encoding for a fluorescence protein, tag protein, antibiotics, or the recognition sequence for a restriction enzyme digestion).

CRISPR/Cas system requires proto-spacer adjacent motifs (PAMs) that are short 2–6 base-pair sequences in the virus genome adjacent to Cas nucleases targeted sequences (Shah et al., 2013). Jinek et al. (2012) discovered that the CRISPR/Cas9 scheme can, *via* cut and paste by programming, guide RNA to target a precise position in the destination genome, to make genome modifications. CRISPR/Cas9 can edit, delete, or deliver new genes as well as knock out or knock-in. The CRISPR/Cas9 gene editing involves the formation of breaks in both strands of the target DNA. As shown in Figure 1, the breaks subsequently trigger one of two DNA repair mechanisms: homology-directed repair (HDR) or non-homologous end-joining (NHEJ). The mechanism of NHEJ is error-prone, occurs in the cells of mammals, and can lead to insertion or deletion of a certain sequence of DNA, subsequently modifying the protein-coding sequences. The HDR mechanism includes homologous recombination with donor DNA sequences in the targeted DNA, such as the insertion of the DNA sequence encoding a reporter gene of green fluorescence protein (Baena-Lopez et al., 2013; Irion et al., 2014; Lin et al., 2014). The use of HDR to create genome editing has encouraged the development of several methods to make DNA breaks in a sequence-precise approaches the zinc finger nucleases (ZFNs), the transcription activator-like effector (TALE)-nucleases (TALENs), the meganucleases, and currently the CRISPR/Cas9 nuclease that is a fast, cheap, accurate and efficient method (Cox et al., 2015; Maeder and Gersbach, 2016).

## Enhancing the Safety and Fidelity of the CRISPR/Cas9 Editing Tools

Nowadays, many methods of incorporation between CRISPR/Cas9 schemes and viral uses have been explored for inherited disease treatments. This could provide simplicity and precision in mammalian genome editing. However, in these investigations, the use of different viral vectors (e.g., lentivirus, adenovirus, and adeno-associated virus) incorporated with CRISPR/Cas9 system delivery have ingrained imperfections, particularly their immunogenicity, not being a good carrier, and slight loading capability, which significantly restricts the clinical translation based on the *in vivo* studies (Wang et al., 2017; Liu et al., 2020). Recently, several reports have revealed that successful rescue of some genetic diseases is caused by mutations through injecting recombinant single guide RNAs (sgRNAs) and AAV9-packaged Cas9 nuclease (Min et al., 2019).

A variety of studies on animals suggested that the high levels of AAV (≥1.5 × 1014 vg/kg) may trigger severe hepatic toxicity (Hinderer et al., 2018), while in other previous studies, it was reported that the efficacy of *in vivo* CRISPR/Cas9 as a versatile genomic editing therapy was highly level reliant relative and that augmented the level of sgRNA AAV relative to Cas9 AAV improved the competence of genomic editing approach (Min et al., 2019; Zhang et al., 2020). One way to overcome this restricted property might be reducing the viral level applied for gene therapy without negotiating the competence of genome editing and to avoid favored reduction of the sgRNA AAV genome. Zhang et al. (2020) believed that the packaged a CRISPR sgRNA expression cassette into a double-stranded AAV vector, which can generate by mutating the terminal resolution spot arrangement on one crosswise of the inverted terminal repeats, would result in the synthesis of self-complementary AAV (scAAV) (Zhang et al., 2020). Recently, the fast advancement of drug delivery systems (DDS) in several biomedical applications supplied innovative chances for enhancing the pantonality uses of CRISPR/Cas9 schemes as a powerful therapeutic issue. Compared with viral carriers, modern DDS generally have high loading capability and low immunogenicity (Zhang et al., 2019; Wei et al., 2020). Furthermore, the DDS technology normally has inferior immunogenicity and elevated loading capability (Zhang et al., 2019; Wei et al., 2020). Moreover, the employ of DDS can deliver the variations of PK and *in vivo* supply through packing the multidrug blend within the identical delivery service. To date, scientists have effectively established several DDS, comprising lipid-based nanoparticles (Luo et al., 2018; Liu et al., 2020), inorganic nanoparticles (Zhang et al., 2019), and polymer-based nanoparticles (Chen et al., 2019; Liu et al., 2020), to concurrently provide Cas9 protein and sgRNAfor CRISPR/Cas9-established diseases therapy (Luo et al., 2018; Liu et al., 2020).

In our previous article, we previously summarized the distinct forms of the CRISPR/Cas9 techniques and base editors (BE), generalized the various approaches produced to distinguish off-target effects, and examined the features and the constraints of these off-target assessment systems (Li et al., 2019). Point mutations are a contributing issue in several heritable disorders, but different conventional genomic editing technologies might only rectify point mutations *via* HDR. Commonly, HDR restoration is ineffective and mainly creates random deletions and insertions at the dsDNA cutoff point. Recently, BE that blends cytosine or adenosine deaminase with CRISPR structures were established to stimulate targeted base modifications of RNA or DNA in several animal genera (Zuo et al., 2020). Previously, our work (Zuo et al., 2019) demonstrated instances of how the off-target effects of BE can be diminished *via* biological-understanding-guided engineering to expand the efficiency of these robust genomic therapies systems for either therapeutic treatments or research achievements.

The earliest invention of base editors (BE1) joined Cas9 (inactivated by mutations of H840Aand D10A) and cytosine deaminase (APOBEC1) in the direction of sgRNA to boost the precise transformation of a base from CG to TA, and have been reported by several authors (Komor et al., 2016; Yang et al., 2017). It has been reported that there were several types of BE generations that have been promoted for enhancing the efficiency of the CRISPR/Cas9 approach. Particularly, the base editing effectiveness of the BE3 scheme was enhanced, and BE3 has been generally utilized for gene editing in embryos of various species. To superior impede endogenous base deletion restoration, another study carried out by Komor et al. (2016) blended another UGI copy into BE3 to build a new generation of BE types, which is termed base editor 4 (BE4). This new BE4 displayed greater editing proficiency thanBE3.

With the rapid advancement for enhancing the efficiency of CRISPR/Cas9, a technique termed genome-wide off-target analysis by two-cell embryo injection (GOTI) was developed to identify off-target mutations *via* editing or alteration of DNA sequence in one blastomere of two-cell mouse embryos utilizing either Bes or CRISPR/Cas9 (Zuo et al., 2019) Interestingly, this worthy work for the first time proved that several *de novo* SNVs are accelerated by BE3. A promising elucidation is that our system, GOTI, discovers the cell population developed from a specific gene-corrected blastomere, while a prior survey applied wide pools of cells for which rectification is fluctuating, resulting in a paucity of indicators for irregular off-targets owing to population averaging (Zuo et al., 2019). The off-target impacts of BEs may be diminished by declining the DNA binding capability of APOBEC1 or by manipulating various editions of cytidine deaminase (Wang et al., 2018). Overall, GOT Innovative technology could be a helpful strategy for investigating off-target effects of several pieces in the DNA sequence without the impediment of single-nucleotide polymorphisms appearing in various individuals.

### Sickle Cell Disease and β-Thalassemia

Sickle cell disease (SCD) and transfusion-dependent β-thalassemia (TDT) are considered the most critical monogenic disorders with serious and possibly life-threatening signs. Moreover, both diseases SCD and TDT are thought the most common globally monogenic disorders, with an annual diagnosis in roughly 300,000 and 60,000 patients, respectively and induced by mutations in the gene of hemoglobin β subunit (*HBB*) in humans (Saraf et al., 2014; Frangoul et al., 2021). Disturbance in the β-globin production caused by mutation of HBB gene results in ineffective erythropoiesis and leads to TDT disorder in children (Cao and Galanello, 2010; Frangoul et al., 2021). Furthermore, SCD is the consequence of a spot in *HBB* mutation that switches glutamic acid with valine at an amino acid situation (Saraf et al., 2014). Polymerization of deoxygenated sickle hemoglobin induces hemolysis, anemia, erythrocyte distortion, irreparable end-organ impairment, painful vaso-occlusive episodes, and a lowered life expectancy (Frangoul et al., 2021). Moreover, excessive abundance of adult hemoglobin encourages robust anemia in patients suffering from the disorder (Lamsfus-Calle et al., 2020). Conversely, persons with natural-appearing mutations in the *HBB* associated or cluster genes neutralize this inconsistency *via γ-globin* transcript and following fetal hemoglobin (HbF) synthesis.

Several attempts have produced considerable trial evidence that HbF reactivation by gene interference of specific transcription regulators and factors could offer potential therapeutic support for β-hemoglobinopathies (Zhou et al., 2010; Wilber et al., 2011; Antony et al., 2018). Both *BCL11A* and *KLF1* are the main regulators engrossed in the process of *γ-* to *β-globin* shifting and the suppression of these genes leads to HbF rehabilitation (Shariati et al., 2016). Numerous preclinical and clinical investigations have been presented for prompting HbF by knocking-down genes implicated in HbF suppression (*BCL11A*and *KLF1*) or interrupting the attachment positions of many transcription components in the *γ-globin* gene (*HBG1/2*). Recently, the therapeutic usage of CRISPR/Cas9 in several disorders has been performed involving inherited diseases (Antony et al., 2018). Modifying HBB mutation with CRISPR/Cas9 is one of the greatest hopeful approaches to alleviate hematopoietic disorders, and it has been effectively performed in patient-derived iPSCs without any residual footprint demonstrated in numerous surveys (Traxler et al., 2016). However, lentiviral gene relocation of *β-globin* demonstrated valuable influences in regarded β-thalassemia patients (Negre et al., 2016). Additionally, the high level of semi-random incorporation sites by lentivirus and the transactivation of the proto-oncogene*HMGA2* gene created security worries for this tactic (Cavazzana-Calvo et al., 2010; Negre et al., 2016). Due to the above-stated explanations, CRISPR/Cas9-mediated gene interruption of unique tools to re-express HbF is a favorable applicable option (Antony et al., 2018).Accordingly, several publications have pursued various hereditary regulators by CRISPR/Cas9 to reactivate HbF expression, resulting in a sincere impact after promoters’ genetic intervention of *HBG1/2, LF1*, and *BCL11A* (Canver et al., 2015; Traxler et al., 2016; Lamsfus-Calle et al., 2020). Additionally, many studies have been conducted concerning ability to potentiality alleviate hematopoietic disorders (Lamsfus-Calle et al., 2020; Frangoul et al., 2021).

To compare the targeting screening of several genes such *BCL11A, KLF1,* and*HBG1/2* by CRISPR/Cas9 for the introduction of fetal hemoglobin pattern, a study was conducted to compare targeting screening of *BCL11A,KLF1,* and *HBG1/2* in parallel for their effect on HbF resurgence and presented safety dimensions by molecular assessments to pick the greatest candidate for clinical transformation (Lamsfus-Calle et al., 2020). Interestingly, these researchers found that*BCL11A* gene is a valuable candidate for helping the gene therapy efficiency of β-hemoglobinopathies, with high HbF reappearance, unchanged gene expression, and no off-targets. Additionally, the above-described *HBG1/2* attitude also generated clinically applicable amounts of HbF with ordinary safety reports, and thus, after further studies, this approach could be thought a favorable choice gene therapy for β-hemoglobinopathies (Lamsfus-Calle et al., 2020). The above comparative assessment exhibited that the *BCL11A* gene is the greatest clinically applicable method while *HBG1/2* could signify a hopeful option for the medication of β-hemoglobinopathies (Lamsfus-Calle et al., 2020).

On the other hand, *KLF1* gene is not an appropriate tactic for clinical interpretation because it impaired gene expression after gene editing. In one recent study, scientists constructed a CRISPR/Cas9 can target the *BCL11A* erythroid-specific enhancer (Frangoul et al., 2021). It has been shown that most alleles (80%) were changed at this locus without making signs of off-target editing. Following the undertaking of myeloablation, it was reported that patients (one with TDT and the other with SCD) obtained autologous CD34+ cells which were modified with CRISPR/Cas9 targeting the same *BCL11A* enhancer (Frangoul et al., 2021).Later, following the patient’s status, authors noticed that both patients exhibited superior concentrations of allelic replacement in blood and bone marrow and boosts in fetal hemoglobin that supplied pan cellularly, transfusion individuality, and removal of vaso-occlusive episodes (in SCD patients) (Frangoul et al., 2021).

Overall, however, the CRISPR/Cas9 approach may characterize a favorable therapeutic attitude to the medication of TDT; further clinical and preclinical studies should be done to demonstrate the safety and efficiency of CRISPR/Cas9 in the long-term.

### Atherosclerosis

Hypercholesterolemia (HC) is characterized by very elevated concentrations of low-density lipoprotein (LDL) cholesterol in the blood. It is accepted that HC patients have an elevated lifetime risk of premature cardiovascular syndromes such as heart stroke and atherosclerosis. Studies have reported that HC is a hereditary disease because of a genetic mutation, and the highly popular form of inherited HC is known as familial hypercholesterolemia (FH) (Defesche et al., 2017). According to several studies on humans, FH is an autosomal dominant hereditary disorder with a gene prescription impact and is triggered by mutations in the intended genes encoding the *PCSK9,* apolipoprotein B (*APOB*), or LDL receptor (*LDLR*), with mutations in each such gene accounting for 1, 5, and 90% of FH cases, respectively (Hobbs et al., 1992; Defesche et al., 2017; Zhao et al., 2020). It has been shown that the *LDLR* gene is a cell surface protein which is highly expressed in hepatocytes and its main regulatory role is to eliminate excess LDL from the circulation. The low expression or mutation of the *LDLR* gene could promote the progression and development of atherosclerotic clots (Defesche et al., 2017). The seriousness of atherosclerosis is directly associated with the activity and level of LDLR in the liver (Sun et al., 1994).

Researchers showed that the CRISPR/Cas9 system for DNA repair demonstrates a favorable new therapy for human disorders caused by genetic defects. It was shown that *LDLR* mutation is detected in singular with HC patients (Sun et al., 1994). In this regard, Zhao et al. (2020) generated an *LdlrE208X* mutant knock-in mice idealistic established on a point mutation in the *LDLR* gene. The *LdlrE208X* mutant in mice, which holds a point-nonsense mutation in the exon 4of the endogenous *Ldlr* gene, without the appearance of LDLR protein transcript in the hepatic cells, created acute atherosclerosis after feeding mice with a high-fat diet (Zhao et al., 2020).

It was reported that when the mutant *LdlrE208X* strain was remedied with CRISPR/Cas9 combined with AAV, the abundance of the LDLR gene was moderately repaired, and the potentiality of the atherosclerosis was ameliorated (Zhao et al., 2020). This study assumed that the use of genomic editing strategy *in vivo via* an AAV-CRISPR/Cas9 approach may introduce a favorable therapeutic attitude to the medication of atherosclerosis while possibly improving the effectiveness of existing lipid-lowering drugs (Zhao et al., 2020). There is a paucity of studies on the uses of CRISPR/Cas9 innovative technology for the medication of patients with familial hypercholesterolemia. So further studies are required for providing a potential therapeutic approach *via* CRISPR/Cas9.

### Retinoschisis

Retinoschisis (RT) is a common X-linked juvenile genetic macular retrogression that affects the vision during the early stage of life, with an incidence of 1 in 5,000–25,000 (Molday et al., 2012). The clinical aspects of RT involved with early vision loss correlated with vitreous hemorrhage, retinal detachment, bilateral foveae, and separation of the inner retinal coat (Tantri et al., 2004). Retinoschisin 1 (RS1) gene is linked with X-linked juvenile hereditary, includes six exons, and encodes a protein that consists of 224 amino acids (Tantri et al., 2004; Li et al., 2007). The RS1 gene is explicated and produced by photoreceptors of the inner and outer retina, as was detected in the retina of mice (Li et al., 2007). Earlier reports have revealed that patients with *RS1* missense mutations Arg213Gln, Arg209His, Arg102Gln, and Asp145His display acute RT attributes in the clinical aspects (Wang, 2006; Li et al., 2007). Lower abundance or mutation in the RS1 gene trigger misfolding and may affect extracellular and intracellular protein aggregation, eventually leading to schism and cystic forms in the retina (Wang, 2006; Yang et al., 2020).

The gene therapy of genetic diseases *via* CRISPR/Cas9 is considered a possible method to induce protective or therapeutic mutations in RT disease. There are several studies that have been implemented for applying CRISPR/Cas9 technology to target the *RS1* gene in animal models (Table1).

A study by Yang et al. (2020) used a carboxylated nanodiamond (ND; 3 nm) as a carrier of CRISPR/Cas9 scheme intended to establish a precise mutation *RS1* c.625 C*>*T. This approach is considered a reliable and biocompatible substance for the transformation of a specific region of DNA at the level of *in vitro* and *in vivo*. After further deep investigation, results demonstrated that such an ND could be effectively internalized by mouse retina cells and hiPSCs and might present the mutation to the *RS1* gene. They demonstrated that ND-based CRISPR/Cas9 delivery method of genome editing has a great capacity for determining *in vitro* and *in vivo* disorder forms of X-linked RS disease (Yang et al., 2020), also be used to recondition *RS1* mutation *in vivo*, and be of conceivable significance for gene treatment. Taken together, the genetically inherited RS can be developed *via* manipulating CRISPR/Cas9 to establish the causal mutations into hiPSCs and distinguish them into the strata of cells in the optic cups. Other studies supposed that it is not realistic to develop CRISPR/Cas9-mediated editing given to individual patients mutations because almost 200 mutation spots in the *RS1* gene have been discovered and connected with XLRS (Chou et al., 2020). To fulfill CRISPR/Cas9- intervened knock-in of the RS1 gene in the retinas of XLRS patients *via* either subretinal or intravitreal injection of the supramolecular nanoparticle would present a revolutionary curative therapeutic strategy. It is of interest that the identical approach should be valid for manipulating other genetic disorders in which specific mutations only function in local tissues.

### Phenylketonuria

Phenylketonuria (PKU), triggered by recessively inherited phenylalanine hydroxylase (PAH) defect, represents one of the widely popular neonatal faults of metabolism (IEMs). PKU is characterized by hyperphenylalaninemia and neurotoxic impacts of phenylalanine upon the brain (Blau, 2016). Additionally, PKU is a monogenic syndrome resulting from the deficiency of PAH enzyme synthesis in liver hepatocytes. The disturbance of biological function of PAH recurrent unremedied hyperphenylalaninemia patients triggers serious neurological impairment, resulting in psychological disorders, intellectual disability, and seizures (Blau, 2016; Cazzorla et al., 2018). Several attempts have been made to manage the PKU disease such as dietary management and restrictions and other therapeutic agents. According to the severity of PKU disorder, it has been reviewed that depressing the level of plasma phenylalanine avoids intellectual disability; sustaining the level in the therapeutic range of 120–360 μmol/L is related to great effect for patients as well as their pregnancies (Cazzorla et al., 2018). However, in recent years with the advancement of gene therapy technology, many investigations have summarized the gene therapeutic attempts of correcting *in vivo* and *in vitro* models for PKU and exhibited the potentiality for using gene therapies such as CRISPR/Cas9 genome-editing for future implications for human gene therapy (Grisch-Chan et al., 2019; Koppes et al., 2020; Richards et al., 2020) (Table1).

By applying CRISPR/Cas9 gene editing in PKU disease, Koppes et al. (2020) established a pig model of traditional PKU that summarizes the biological and brain outcome observed in untreated human patients with PKU, recognizing it as a powerful template for upcoming clinical experiments. Koppes et al. (2020) developed PAH-null pigs as a preclinical animal model for PKU disease to examine pathological aspects and assess innovative therapeutic mediations. In this study, the zygote was injected with Cas9 mRNA and two sgRNAs that showed losses in preimplantation embryos, with embryo transfer to a replacement leading to two creator pigs. Results exhibited that the pig model was multiple heterozygous for losses of exon 6 and 7, while one animal was heterozygous for a PAH exon 6 removal allelic affects. The treated animal model presented hyperphenylalaninemia (2–5 mM) which was remediable by dietary Phelimitation, and steady with traditional PKU, along with hypopigmentation, ventriculomegaly, growth delay, and reduced brain gray matter size.

In another study, Richards et al. (2020) reported that CRISPR/Cas9 technology exhibited incessant rectification of the Pahenu2 allele in a part of treated hepatic cells of mice with PKU, creating considerable decrease of blood phenylalanine, yielding partial restoration of liver PAH activity, and avoiding maternal PKU effects during pregnancy. As stated above, these efforts reveal that CRISPR/Cas9 technology could be used as a confident device for constant PKU gene editing for more in-depth exploration to treat genetic disease.

The limitations of using CRISPR/Cas9-intervened gene editing is that a distinct guide RNA—and, hence, one of the two AAV vectors—would have to be remodeled for every single patient with a unique mutation, or at least they would require distinctive components for each exon of the *Pah* gene. Gene modification for PKU patients is a favorable innovative method to boost lifetime neurological safety while permitting unhindered nutritional phenylalanine consumption by designing liver tropic recombinant AAV2/8 vectors to make CRISPR/Cas9 available system for PKU syndrome.

### Muscular Dystrophy

Muscular dystrophy (MD) is described as the progressive destroying of skeletal muscles development that is caused by genetic or spontaneous inherited mutations. Duchenne muscular dystrophy (DMD) is amongst the most widespread of human heritable syndromes, influencing roughly 1:5,000 newborn males (Mendell et al., 2012). Dystrophin gene mutations (DMD) cause the defeat of the presence of both dystrophin and dystrophin-glyocoprotein convoluted, triggering sequences of regeneration and necrosis, muscle membrane fragility, and cumulative muscle weakening (Batchelor and Winder, 2006). A range of methods for genomic therapy medications of DMD is in advancement, many of which benefit from the efficiency of vectors derived from AAV to provide genes systemically *via* the vasculature (Bengtsson et al., 2015).

CRISPR/Cas9 is the most promising gene editing approach involving AAV vectors for treating DMD. By directing the mutational hotspot at exons 45–55 and establishing changes within exons or erasing one or more exons, the trial investigated by Ousterout et al. (2015) intended single or multiplexed sgRNAs to repair the dystrophin reading frame in humans. Subsequent genomic editing in DMD patient myoblasts, with restoration of dystrophin genes, has been identified in an *in vitro* trial. Furthermore, human dystrophin was also discovered *in vivo* after transplantation of genetically modified patient cells into immune-deficient mice. Remarkably, the distinguishing multiplex genomic editing services of the CRISPR/Cas9 platform technology empower the synthesis of a single significant erasure that can rectify up to 62% of DMD mutations (Ousterout et al., 2015). From this, Bengtsson et al. (2017) developed many tactics for genomic editing, specifically the mutation in dystrophic *mdx4cv* mice *via* single and dual AAV vector/Cas9supply incorporated with a sgRNA cassette to fully rectify the mutation of dystrophin homology district in mice. It has been reported that the muscle restricted by Cas9 technology appearance facilitates precise gene-editing of the intent mutation, multi-exon removal, or complete gene rectification using HDR method in myogenic cells. In the same context, Zhang et al. (2020) reported that high doses of AAV are needed for effective *in vivo* genome editing *via* packaging of Cas9 nuclease in single-stranded AAV (ssAAV) and sgRNAin self-complementary AAV (scAAV) and provided this dual AAV scheme into a mouse as an animal model of DMD. Consequently, the high level of scAAV also necessary for effective genome editing was at least 20-fold lesser than with ssAAV. This study reported that mice that received systemic therapy indicated recuperation of dystrophin appearance and enhanced muscle contractility in mice. It seems that the effectiveness of CRISPR/Cas9–intermediated genomic editing technology can be significantly enhanced *via* incorporation with the scAAV system. This characterizes an essential development for therapeutic application and translation of genome editing for patients of DMD.

In another recent research that was implemented on dogs as an animal model, Mata López et al. (2020) used CRISPR and TALEN to rejuvenate the expression of dystrophin *via* HDR in myotubes/myoblasts and later *via* intramuscular injection of golden retriever muscular dystrophy dog.

In an *in vitro* experiment, it has been documented that both RNA and DNA sequences were effectively rectified in mice, although dystrophin protein was not transformed. The same research assumed that with intramuscular injection of both distinct guide arms, sgRNA A and B, the mRNA of dystrophin has been expressed.

The expression of dystrophin reached up to 6% of regular amounts and has been identified in two animals injected with sgRNA B and up to 16% of regular level in one animal subjected to sgRNA treatment. Moreover, the TALEN-treated group did not exhibit restoration based on the expression of dystrophin. Lek et al. (2020) presented a genome-wide CRISPR/Cas9 screen to discover genes whose loss-of-function presented survival when DUX4 was detected in muscle cells. Treatment of hypoxia signaling inhibitors caused in boosted DUX4 protein turnover and following decrease of the cellular hypoxia action and cell loss. In addition, these components demonstrated profitability in lowering facioscapulohumeral muscular dystrophy (FSHD) syndrome biomarkers in patient’s myogenic lines, as well as enhancing functional and structural features in zebrafish models of FSHD. The potentiality of therapeutic advantage for FSHD introduces an augmented paradigm toward mechanistic comprehension and therapeutic detection of a multifaceted heritable syndrome, which may be changeable to other disorders with well-recognized phenotypic choice attempts. Even though attempts in several therapeutic tactics have been made to date, the medications presented for DMD remain supportive and mitigative to enhance the signs of the disorder, rather than therapeutic.

To further improve the editing efficiency of gene editing for spinal muscular atrophy (SMA) patients, Lin et al. (2020) successfully implemented splicing correction of exonic splicing silencer (ESS)-A and B of survival motor neuron (SMN2) gene exon 7 *via* base editing, and thus accomplished an effective and identical A36G transformation. The physiological role of the SMN protein generated by the A38G and A36G synchronized transformation was initially validated by *in vitro* apoptotic attempts; however, the *in vivo* impact still entails more exploration (Lin et al., 2020). The prior work may present a proof-of-rule survey that demonstrates a modern therapeutic approach for remediation of SMA patients using the base editing-mediated splicing rectification *via* BE.

### Cystic Fibrosis

Cystic fibrosis (CF) is a commonly lethal hereditary syndrome that affects the lung and digestive tract and is triggered by mutations of cystic fibrosis transmembrane conductance regulator (CFTR) gene, situated on chromosome 7 (Hart and Harrison, 2017; Marangi and Pistritto, 2018). Given the morbidity for CF, it has an occurrence of 0.0004 live births with a prevalence in those of northern European descent (Marangi and Pistritto, 2018). The CFTR gene has a critical role in regulating the ion channel and osmotic regulation in the body, which makes an adenosine triphosphate (ATP) attach with chloride anion channel. Also, it could permit the modulation of excretion of both molecules such as bicarbonate and chloride.

In the lungs, stem cell functions have been discovered, along with so-termed bronchioalveolar stem cells in the hospital. Moreover, there may be potential to acquire stem cells derived from lungs of CF patients, which were engineered using CRISPR/Cas9 innovative technology to rectify the mutation of CFTR and reinsert them into one of those lung niches where stem cells get their appropriate microenvironment for their growth, development, and survivability (Firth et al., 2015).

Several experiments have been performed to improve the survivability of CF patients by using CRISPR/Cas9 technique (Schwank et al., 2013). Hopeful uses of CRISPR/Cas9 system are also presented on CF and other types of inherited disease in Table 2. Schwank et al. (2013) exhibited the complete reestablishment of CFTR protein functionality utilizing the approach in the shape of cultured stem cells obtained from intestinal CF pediatric patients.

**TABLE 2:** CRISPR clinical trials for some inherited disorders recorded in the current review.

Disease	Target gene	Model	Findings	References
β-thalassemia	*BCL11A*	patients with TDT and SCD	▪ Patients had high levels of allelic editing in	Frangoul et al. (2021)
			▪ bone marrow and blood, increases in fetal hemoglobin that were distributed pancellularly, transfusion independence, and (in the patient with SCD) elimination of vaso-occlusive episodes	
Recessive dystrophic epidermolysis bullosa	*COL7A1 *	patient keratinocytes	▪ This *ex vivo* non-viral approach rendered a large proportion of corrected cells producing a functional collagen VII variant	Bonafont et al. (2019)
			▪ The effective targeting of the epidermal stem cell population enabled long-term regeneration of a properly adhesive skin upon grafting onto immunodeficient mice	
			▪ A safety assessment by next-generation sequencing (NGS) analysis of potential off-target sites did not reveal any unintended nuclease activity	
Duchenne muscular dystrophy (DMD)	*dmd*	human patient with DMD–derived iPSCs	▪ Correction of exon 44 deletions through exon skipping or reframing of surrounding exons could potentially treat ∼12% of patients with DMD	Min et al. (2019)

Furthermore, rectification of the mutation of CFTR gene has been achieved in iPSCs *via* CRISPR/Cas9 gene editing tool (Crane et al., 2015). Using CFTR/Cas9 gRNA vector, it has been presented that iPSCs were acquired by reprogramming somatic skin fibroblasts obtained from CF patients into an embryonic stem cell state, consequently distinguished in the direction of a proximal airway epithelial cell (Crane et al., 2015).

Sheep may be a mainly applicable animal to model CF in humans owing to the resemblances in lung development, size, anatomy, and the length of the gestation in the two genera (Fan et al., 2018), which permits for long-term *in utero* reflections. For these reasons, the ovine fetus has been widely employed for developing cutting edge perinatal therapies. In a study focused on the sheep model, Fan et al. (2018) generated cells with *CFTR* gene disturbance and employed them for the construction of *CFTR–/–* and *CFTR+/–* lambs. It was reported that the newborn *CFTR–/–* sheep established acute disorder in harmony with CF pathology in humans. Of particular relevance were an intestinal impediment, pancreatic fibrosis, and dearth of the vas deferens. Likewise, the significant liver and gallbladder disease may suggest CF liver disorder that is obvious in humans. The phenotype of *CFTR–/–* sheep indicates this huge animal model will be a valuable resource to develop the progress of new *CF* gene editing therapeutics. Likewise, the production of specific human CF disorder–correlated mutations in sheep may develop differentiated medicine for this universal inherited disease. It seems that the convenience of an ovine model of CF will open new prospects to explore the early disease development, which is difficult to research in humans, and could lead to the development of innovative therapeutic approaches (Fan et al., 2018).

Studies have demonstrated the CRISPR/Cas9-mediated production of CF rabbits, a model with a relevant long lifespan and reasonable conservation and care prices (Xu et al., 2021). The CF rabbit as a feasible animal model presents the CF study community as an intermediate-sized model that is related to human CF pathogenesis and treatment. Iranian researchers have explored the genetic modification of CF with ^Δ^F508 mutation of the *CFTR* gene using CRISPR in peripheral blood mononuclear cells (PBMC) (Khatibi et al., 2021).

In this study, they tested a sgRNA-Cas9 plasmid to target the CFTR gene. Results have shown the feasibility of site-specific gene targeting with the CRISPR/Cas9 system. Moreover, 33% of the models were rectified using CRISPR in mutant locus and confirmed by sequence blast at NCBI databases and primers outside the arm locus. So, it could be said CRISPR/Cas9 attitude symbolizes an effective implement to restore the ^Δ^F508 mutation of the CFTR gene in PBM (Khatibi et al., 2021). In one recent study, Vaidyanathan et al. (2021) utilized CRISPR/Cas9 and two AAV holdings with two halves of the CFTR cDNA to consecutively insert the complete CFTR cDNA alongside a truncated CD19 (tCD19) enrichment tag in upper airway basal stem cells (UABCs) and human bronchial basal stem cells (HBECs). The altered cells were enriched to achieve 60–80% tCD19^+^ HBECs and UABCs from several distinct CF donors with a diversity of mutations. Distinguished epithelial monolayers cultured at air-liquid interface showed restored CFTR function that was >70% of the CFTR function in non-CF controls. Geurts et al. (2020) demonstrated that CRISPR-9 based adenine can be efficiently suitable in human adult stem cells, accentuating its clinical possibility and applicability in the genetic restoration of cystic fibrosis. Hence, it seems that the application of CRISPR/Cas9 potentially empowers the progress of a gene therapy medication for almost all CF patients, involving patients who cannot be treated by applying lately accepted modulator remedies.

### Dystrophic Epidermolysis Bullosa

Dystrophic epidermolysis bullosa (DEB) is an inherited skin syndrome triggered by mutations to the kind VII collagen gene (*COL7A1*), located on chromosome 3, that deactivates a structural of protein synthesis and is a vital player for skin straightness (Webber et al., 2016).

The subsequent failure of the functional site of this targeted protein (C7) at the dermal-epidermal junction concedes the completeness of the attachment of the epidermis to the dermis, resulting in acute fibrosis, scorching, and a susceptibility to squamous cell carcinoma. A variety of therapeutic attempts to reinstate *COL7A1* expression have been depicted for the treatment of RBEB (Shen et al., 2014; Webber et al., 2016).The research of Webber et al. (2016) examined using the CRISPR/Cas9 scheme to enable rectification of an RDEB triggering the mutation of *COL7A1* gene in primary fibroblast cells that were then applied to develop iPSCs. Results have revealed that CRISPR/Cas9 is an adjustable genomic editing approach that can be paired with iPSC innovative to generate multiple gene-rectified autologous cell forms with therapeutic conceivable for RDEB patients. In an *ex vivo* gene therapy trial**,** a study by Hainzl et al. (2017) applied the CRISPR tool to rectify an extremely frequent homozygous mutation in COL7A1 exon 80 (6527insC).

In this study, it has been reported that the introduction, with findings in a premature termination codon, accounts for 46% of alleles in the Spanish population that have DEB disorder (Schwarz et al., 2014). Hainzl et al. (2017) used either Cas9or spCas9 as a gene editing tool for RDEB keratinocytes, incorporated with a related granter model for HDR stimulation, which resulted in phenotypic rectification as proven *in vivo* and *in vitro* trials in a xenograft mice model. Collectively, the possible uses of the CRISPR/Cas9 system have been achieved for the precise *ex vivo* rectifying of a persistent mutation of *COL7A1* leading to the dystrophic form of EB in mice (xenograft model). Utilizing a dual sgRNA-guided Cas9 nuclease delivered as a ribonucleoprotein complex *via* electroporation, Bonafont et al. (2019) realized significant effective pointed removal of COL7A1 exon 80 in RDEB patient keratinocytes concerning a very ordinary frameshift mutation. Moreover, Takashima et al. (2019) used CRISPR/Cas9 targeting a recurrent frameshift mutation (c.5819delC) in COL7A1gene to RDEB patient fibroblasts. This study found a substantial 34% of clones in which the frameshift in a mutation of COL7A1 was repaired. Additionally, the repairing of *COL7* gene was purposeful, as illustrated by triple-helix creation for *in vitro*, and was accurately delivered in the basement membrane zone in mice. Mutation site-precise non-homologous end-joining might be a highly effective gene remedy for hereditary syndromes triggered by frameshift mutations (Takashima et al., 2019). End-joining‒based gene editing is commonly utilized for effective reframing and knockout of target genes. Recently**,** a study has shown that the use of COL7A1 editing centered on specific end-joining‒mediated DNA restoration is an effective approach to restore the disorder-connected nature of DEB irrespective of the mutational inheritance (Kocher et al., 2020). Based on these results, this *ex vivo* gene therapy attitude may have the ability to be modified for clinical purposes in the upcoming era.

### Ornithine Transcarbamylase Deficiency

Ornithine transcarbamylase (OTC) deficiency is an infrequent X-linked inherited disorder described by partial or complete absence of the enzyme OTC. The main biological function of OTC is participating in the regulation of break down and removal of nitrogen in the body *via* the urea cycle (Lichter-Konecki et al., 2016). It has been revealed that the inadequacy of the OTC enzyme synthesis in humans triggers frequent and life-frightening events of hyperammonemia (Batshaw et al., 2014; Lichter-Konecki et al., 2016). In males, hemizygous has the deficiency of OTC, the first metabolic disaster generally appears in the neonatal phase and is related with up to 50% mortality, with survivors normally suffering liver metabolic disorder in the initial stages of their life (Ah Mew et al., 2013). In mice, Yang et al. (2016) established an approach *via* a dual-AAV system based on AAV8, which has superior hepatic tropism of OTC, to rectify the spot of OTC mutation in neonatal *spfash* mice utilizing CRISPR/Cas9 strategy.

Researchers used intravenously intervention of two AAVs, one conveying Cas9 and the other expressing a donor DNA and the guide RNA, into a neonatal mouse (as an animal model) with an incomplete weakness in the uric phase syndrome of enzyme OTC (Yang et al., 2016). Results indicated that degeneration of the mutation in 10% of hepatic cells enhanced the survivability of neonatal mice subjected to a high-level of protein in the diets, which aggravates OTC disorder. It was demonstrated that gene editing in adult OTC-deficient mice was inferior and attended by greater erasures that ablated residual appearance from the endogenous *OTC* gene, producing a reduction in the tolerance to protein and deathly hyper ammonemia on a food diet (Yang et al., 2016).

Numerous earlier investigations have struggled to insert a serviceable version of the CFTR cDNA utilizing viral and non-viral approaches both *in vivo* and *in vitro* (Cooney et al., 2016). In these statements, the appearance of CFTR protein was powered by exogenous exciters and the CFTR abundance feature was not introduced in the endogenous CFTR locus. However, according to the previous reports on CF of human and pig models, it was noticed that renovation of CFTR protein services subsequent gene alternatives (Tomco et al., 2016).

The advancement of CRISPR/Cas9 as a hopeful genomic editing tool allows the incorporation of the CFTR cDNA in the endogenous locus and hence should facilitate the protection of indigenous CFTR expression, contained in monocytes and ciliated cells. In a recent research, Wang et al. (2020) found that a CRISPR/Cas9 technology homology-guided restoration can rectify a G-to-A mutation in hepatic cells of neonatal OTC mice in nearly 10% of OTC alleles. Consequently, this CRISPR/Cas9 technology may be relevant to all patients with deficiency of OTC, regardless of mutation and/or clinical condition (Wang et al., 2020). One limitation has been demonstrated in this study, in which an editing vector capable of rectifying one mutation would not be appropriate for patients holding various OTC mutations, plus the expression would not be quickly sufficient to remedy a hyperammonemia disorder.

### Primary Hyperoxaluria Type 1

Primary hyperoxaluria (PH) is a cluster of inherited metabolic diseases that are characterized by the aggregation of a material recognized as oxalate through defects in enzymes complicated with glyoxylate metabolism in the kidneys and other tissue structures of the body (NORD, National Organization for Rare Disorders). The overproduction of oxalate (an end-product of glyoxylate cycle in the kidney) is the main cause of kidney damage for PH in humans (Salido et al., 2012; Cochat and Rumsby, 2013). Normally, oxalate is created at minimal amounts and constantly eliminated by the kidneys from circulation and emitted in urine. Otherwise, renal aggregation of oxalate leads to nephrocalcinosis and urolithiasis and progresses to end-stage renal disease (Cochat and Rumsby, 2013). Reports indicated that there are three types of PH (PH1, 2, and 3), with PH1 being the most common (around 70–80% of all PH patients) and serious subtype (Edvardsson et al., 2013; Hopp et al., 2015). One assessment places the incidence of PH type I at 1–3 cases per 1,000,000 people in the overall population with fewer than 1,000 individuals with PH in the United States and the prevalence at 1 case per 120,000 live births per year in Europe. Likewise, PH disease is assumed to be roughly 2.5 times more common in European Americans than African Americans (source, NORD). Genetically, PH1 is a life-threatening syndrome caused by the *AGXT* gene mutation, resulting in decreased activity of hepatic enzyme alanine-glyoxylate aminotransferase (AGT), which catalyzes glyoxylate conversion to glycine (Hopp et al., 2015; Zabaleta et al., 2018). The malfunction of *AGT* gene or proteins leads to a cumulative reduction of glomerular filtration rate, and finally, results in ESRD and, if untreated, mortality in high patients (Zabaleta et al., 2018). Many previous investigations have been developed an Agxt−/− mouse model using CRISPR/Cas9-facilitated genetic material editing for the clinical phenotype of PH1 patients and may be a useful tool in developing novel therapies for this devastating disease (Zheng et al., 2018; Li et al., 2021).

Several types of researches have aimed to discover more appropriate remedies with lower side consequences to treat PH1; CRISPR/Cas9 scheme presents novel ways for the advancement of innovative treatments for sundry unmet clinical requests, counting a substantial number of hereditary monogenic disorders such as PH1 (Zabaleta et al., 2018; Estève et al., 2019; Zheng et al., 2020). One method is using a developed substrate reduction therapy (SRT) for PH1 constructed on CRISPR/Cas9-mediated genomic editing tool. A study by Zabaleta et al. (2018) reported that single systemic management of an AAV8-CRISPR/Cas9 vector for affecting glycolate oxidase impedes the overproduction of oxalate as well as induces kidney impairment, with no symptoms of poisoning in *Agxt1*−/− mice**.** Findings show that renal oxalate aggregation is stopped in mice treated with CRISPR/Cas9 and that they were fully safeguarded versus metabolic defiance with an oxalate precursor, demonstrating lowered oxalate crystal synthesis in the kidneys and lowered oxalate secretion in urine (Zabaleta et al., 2018).

This study has presented only one side-impact; when editing *Hao1* there was a substantial boost of glycolate quantities in urine, which is thought to be a non-pathological sign. Comparable outcomes were earlier described in mice inadequate in both *Hao1*and *Agxt*, and mice treated with *Hao1* siRNA (Martin-Higueras et al., 2016).

Estève et al. (2019) reported the CRISPR/Cas9 nuclease intermediated gene targeting of a single-copy *AGXT* gene restorative minigene into the safe harbor of AAVS1 locus in PH1-caused PH1-iPSCs without off-target insertions. They found a vigor post transcription of a codon-adjusted AGT in HLCs derived from AAVS1 locus-edited PH1-iPSCs. This research offers the impervious perception that CRISPR/Cas9-intermediated incorporation of an *AGXT* minigene into the AAVS1 safe harbor locus in patient-precise iPSCs is an effective way to produce functionally rectified hepatic cells, which in future explorations may assist as a source for autologous cell-based gene therapy for the medication of PH1. It seems that findings reveal the feasibility of repairing *AGT* expression *via* the uses of CRISPR/Cas9 as a versatile genomic editing and cell reprogramming approach for PH1 patients.

Another critical point of view for PHI patients is that lactate dehydrogenase (LDH) is a potential model target for weakening oxalate production as it is in charge of glyoxylate to oxalate conversion in the hepatocytes, the latter phase of oxalate metabolism. Zheng et al. (2020) examined the therapeutic efficiency and probable side impacts of CRISPR/Cas9 capability to manage PH1 *via* precisely interrupting the hepatic *LDH* gene. Findings exhibited that the *Ldha* gene was particularly knocked out in 20% of the hepatocytes of PH1 rats in the therapy group, resulting in 50% more reduction in the expression of the LDH than that in the untreated group. Moreover, the use of CRISPR/Cas9 technology targeting *Ldha* gene demonstrated that urinary oxalate concentrations were substantially diminished, and renal calcium oxalate precipitation was principally alleviated in the CRISPR/Cas9 group during the whole experimental period (around 6 months) (Zheng et al., 2020). Additionally, it was shown that no CRISPR/Cas9-associated off-target edits or hepatotoxicity was discovered in the previous work. Obviously, CRISPR/Cas9 may be a new therapeutic applicable scheme for mitigating PH1 for its long-lasting impact *via* LDH disruption and low editorial efficiency requirements. The high editing efficiency of the system is translated into an outstanding therapeutic effect.

### Hereditary Hearing Loss

Hereditary hearing loss (HHL) is a neurosensory and inherited syndrome caused by monogenetic or polygenic disorders that affects every 1/500 infants globally and almost 1/3 people over the age of 65 years (Farooq et al., 2020). The use of gene therapy to remedy HHL is a suitable and versatile strategy since it can intent defective molecular modules of auditory transduction to reestablish and repair the function of regular cochlear. Several efforts have been conducted to design CRISPR/Cas9 which is sustainable to investigate ear cell heredities by constructing gRNA giving to selective gene **(** Akil et al., 2012; Mianné et al., 2016). Considering the previous research on hearing loss, *in vivo* gene rectification of the inner ear *via* HDR-mediated renovating has a slight improvement because of the inferior effectiveness of HDR (< 1%) as compared with NHEJ (> 85%) (Bischoff et al., 2020). Enhancing its efficiency is the first criterion for its broad use (Mali et al., 2013a). By using some vertebrate animal models, human hearing loss genes have been broadly explored or screened, such as myosin VIIA, Xin-actin binding repeat containing 2, transmembrane channel–like protein 1, calcium and integrin-binding protein 2,leucine-zipper, epiphycan, cadherin 23, and sterile-alpha motif kinase Zak (Akil et al., 2012). CRISPR/Cas9 gene editing is utilized on a different animal model to reduce the mutation intensity and to increase awareness of the disorder pathology. The following human deafness genes have been successfully edited using the CRISPR/Cas9 technology to regain hearing. CRISPR/Cas9 has been effectively used in model animals to edit hearing genes (Akil et al., 2012).

Several studies have been performed to treat HHL using CRISPR/Cas9 depending on the target gene such as transmembrane channel–like protein 1 (Zuris et al., 2015) leucine-zipper containing kinase (ZAK) (Kraft et al., 2015), calcium- and integrin-binding protein 2 (CIB2) (Wang et al., 2017), and adhering 23 (CDH23) (Mianné et al., 2016). An *in vivo* study has been conducted using delivery of a single Cas9-Tmc1-mut3-lipid injection into the inner ear of Tmc1Bth/+ mice to remedy autosomal dominant developing hearing loss concerning hair cell impairment by deactivating mutant allele. After several weeks, hair cells seemed healthy in these mice **(** Zuris et al., 2015). CRISPR/Cas9-mediated the removal of two ZAK isoforms achieved *via* single gRNA in exon 2 and transfection of CRISPR structure into mouse embryonic stem cells. Subsequently, Kraft et al. (2015) mentioned that this potent mutation is lethal for mice embryos, while SAM domain deletion *via* CRISPR/Cas9 system finally stimulated inferior expression of SHFM genes such as Trp63 and complicated hind limb defect/HHL. Further validation for demonstrating the plausible association of those genes and auditory function, CIB2 and CIB1 genes in KO mice are created *via* the CRISPR/Cas9 technology (Wang et al., 2017). They found that the deletion of the CIB2 gene led to acute deafness and prevented mechanoelectrical transduction flows in auditory hair cells, while deletion of CIB1 gene did not induce auditory function in mice (Wang et al., 2017). Microinjection of CRISPR/Cas9-mediated HDR was reported at zygotes of inherited C57BL/6NTac mice, in which Cas9 (D10A) nickase enzyme combined with single-stranded oligonucleotide donor and gRNA has been employed to effectively restore defected alleles. It repairs a faulty gene’s genotype, thus restoring their auditory function phenotypically (Mianné et al., 2016). Providing Cas9 DNA technology could achieve superior genome editing competence, although prompt a retentive delay time to make the appearance, superfluous transgene results affecting more off-target effects, and possibly greater immunogenic reaction.

## Conclusion

CRISPR/Cas9 has appeared as a persuasive new technology for manipulating and visualizing genomes to perform precise genome editing. In the existing review, we highlight the understanding of CRISPR/Cas9 biology as well as the strategies for enhancing the efficacy of the CRISPR/Cas9 editing tool. CRISPR/Cas9 approach has been employed in a variety of fields including disease therapy associated with genetic disorders. However, several limitations and challenges remain and should be mitigated. Future exploration should refer some constraints, containing the effects of long-term expression of Cas9 nuclease *in vivo*, the longevity of intended gene abundance, and possible immunological reactions to nuclease as well as precise protein. Before the use of CRISPR/Cas9 for human disorder rectification, attempts were made to enhance and maximize the editing competence as well as reduce off-targets and develop modern devices to precisely deliver the CRISPR/Cas9 modules to the target tissue for gene editing (Zuo et al., 2019; Zuo et al., 2020). The potential uses of CRISPR/Cas9 technology will provide healthier lives for people by alleviating complicated diseases. Future surveys should assess this innovative tool in larger numbers of animals and, finally, patients with inherited diseases, so other investigations are required before translation to the clinic. Furthermore, there is a need for further *in vivo* surveys scrutinizing the off-target effects caused by the medication by gene therapy and the prospective immune responses stimulated by viral delivery vectors and more responsive analyses to alleviate the possibility of immunogenicity. Ultimately, according to the findings of the first long-period report, upcoming preclinical trials should be concentrating on boosting competence and augmenting the proportion of the intended gene modulations by enhancing delivery and gene editing approach.
